# Governing Ecological Connectivity in Cross-Scale Dependent Systems

**DOI:** 10.1093/biosci/biab140

**Published:** 2022-01-25

**Authors:** Annika T H Keeley, Alexander K Fremier, Pascale A L Goertler, Patrick R Huber, Anna M Sturrock, Samuel M Bashevkin, Blake A Barbaree, J Letitia Grenier, Thomas E Dilts, Melanie Gogol-Prokurat, Denise D Colombano, Eva E Bush, Angela Laws, John A Gallo, Mathias Kondolf, Amanda T Stahl

**Affiliations:** Delta Stewardship Council, Sacramento, California, United States; Washington State University; Delta Stewardship Council, Delta Science Program, Sacramento, California, United States; University of California, Davis, Davis, California, United States; University of Essex, Colchester, England, United Kingdom; Delta Stewardship Council, Sacramento, California, United States; Point Blue Conservation Science, based Petaluma, California, United States; San Francisco Estuary Institute, Richmond, California, United States; University of Nevada, Reno, United States; California Department of Fish and Wildlife's Biogeographic Data Branch in Sacramento, California, United States; University of California, Berkeley, Berkeley, California, United States; Delta Stewardship Council Delta Science Program, Sacramento, California, United States; The Xerces Society, Portland, Oregon, United States; Conservation Biology Institute, Corvallis, Oregon, United States; University of California, Berkeley, Berkeley, California, United States; Washington State University, Pullman, Washington, United States

**Keywords:** ecological connectivity, cross-scale dependent systems, San Francisco Estuary, governance, restoration

## Abstract

Ecosystem management and governance of cross-scale dependent systems require integrating knowledge about ecological connectivity in its multiple forms and scales. Although scientists, managers, and policymakers are increasingly recognizing the importance of connectivity, governmental organizations may not be currently equipped to manage ecosystems with strong cross-boundary dependencies. Managing the different aspects of connectivity requires building social connectivity to increase the flow of information, as well as the capacity to coordinate planning, funding, and actions among both formal and informal governance bodies. We use estuaries in particular the San Francisco Estuary, in California, in the United States, as examples of cross-scale dependent systems affected by many intertwined aspects of connectivity. We describe the different types of estuarine connectivity observed in both natural and human-affected states and discuss the human dimensions of restoring beneficial physical and ecological processes. Finally, we provide recommendations for policy, practice, and research on how to restore functional connectivity to estuaries.

Ecosystem management and governance of cross-scale dependent systems require integrating knowledge about ecological connectivity in its multiple forms and scales, because, in such systems, ecological processes at one scale depend on connectivity among the different scales (figure 1; e.g., Thrush et al. 2013a). Governance includes any governmental or nongovernmental (i.e., formal and informal) entities involved in “organized efforts to manage the course of events in a social system” (Burris et al. 2008). Ecological connectivity—the flow of organisms and materials across space and time—is important in marine, freshwater, and terrestrial systems; however, the literature does not adequately synthesize the social–ecological connectivity concept to support a cross-scale understanding of the multiple aspects of connectivity, which is essential for ecologically successful governance of complex boundary systems (but see Belote et al. 2020). We term the merger of connectivity concepts across systems and scales ecoscape connectivity. Ecoscape (analogous to ecosystem; see Lidicker 2008) refers to an ecological system (terrestrial, freshwater, or marine) that is composed of two or more types of biological communities and contains both biotic and abiotic components. Ecoscapes can occur at a wide range of spatial and temporal scales.

**Figure 1.**
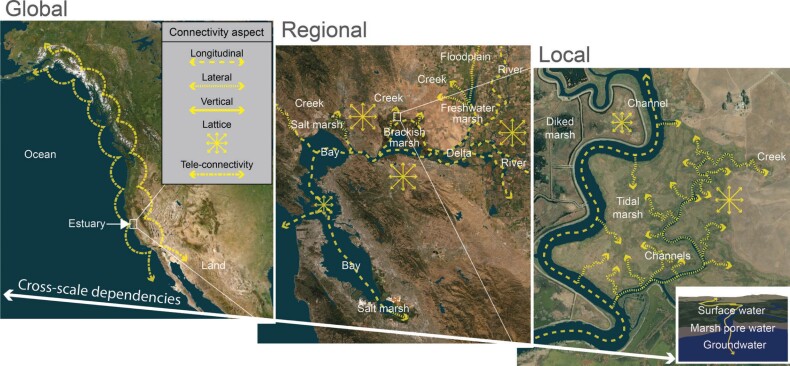
Multiple aspects of ecological connectivity are essential for cross-scale dependent systems. Together, the different aspects of connectivity across realms and scales combine to define ecoscape connectivity.

Two definitions emerge across disciplines for connectivity across the land–sea interface: ecological connectivity—the movement of populations, individuals, genes, gametes and propagules between populations, communities, and ecoscapes and that of nonliving material from one location to another (Hilty et al. 2020) and hydrological connectivity—the water mediated transfer of matter, energy, and organisms within or between elements of the hydrological cycle (Pringle 2001). The terms are not mutually exclusive but have different foci. Hydrological connectivity emphasizes the functional role of water in the biophysical processes that create and maintain the geomorphic system and affect water quality and biogeochemistry and in the embedded processes that shape ecological communities. Ecological connectivity emphasizes the movement of biota and materials as a process that influences population persistence, including the functional connections across landscapes that can be impeded by habitat modification and fragmentation.

Scientists, managers, and policymakers are increasingly recognizing the importance of connectivity for ecosystem functioning. Disruptions in connectivity alter the movements of organisms and nonliving materials and change ecological processes such as pollination, seed dispersal, and disturbance regimes. These disruptions can fundamentally alter the biophysical processes, independently of whether they occur within or outside the focal system (i.e., cross-scale dependence; Hilty et al. 2019). Although loss of connectivity has received the most attention in the literature, an increase in connectivity can also negatively influence ecosystem function (Haddad et al. 2014, Crook et al. 2015, Fletcher et al. 2016). For example, establishing connectivity between formerly isolated populations can cause the loss of genetic variants (Allendorf et al. 2001). Human transportation systems, as well as the transport of freshwater to mitigate water supply shortages, cause the introduction of nonnative, potentially invasive species (Leuven et al. 2009, Crook et al. 2015). Reservoir water releases during dry periods can result in permanent hydrological connectivity in formerly ephemeral streams, which can affect metapopulation dynamics and community composition (Bond et al. 2010). Connecting naturally circuitous unmodified channels via canals results in overconnectivity and can homogenize species composition and reduce local biodiversity (Rahel 2007).

During rapid environmental change, maintaining and restoring natural connectivity (i.e., connectivity similar to historic conditions, neither overconnected nor fragmented) is essential to give wildlife species the best chance of adapting successfully (Vos et al. 2008). With natural connectivity restored, species can shift their ranges in response to climate change (Keeley et al. 2018, Melbourne-Thomas et al. 2021), and viable population sizes and ecological gradients can promote genetic diversity (Sgrò et al. 2011). Advances in methods to estimate where the restoration of natural connectivity will be most beneficial for ecosystems has the potential to guide environmental management and conservation actions (McRae et al. 2012).

Ecosystems with strong cross-boundary dependencies can be difficult to manage effectively through formal governmental structures alone (Woodhouse and Muller 2017). Governmental organizations in many regions of the world are composed of nested units (e.g., country, state, county), often with overlapping jurisdictions (Stahl et al. 2020), and jurisdictions rarely align with watersheds or ecoregions (Young 2006, Folke et al. 2007). Compounding this, terrestrial, freshwater and marine realms are often managed by separate (and often multiple, spatially overlapping) agencies or governmental organizations, some of which must balance decisions for multiple uses (Cash et al. 2006). Therefore, governing ecological and hydrological connectivity requires intentional coordination among governmental organizations and beyond jurisdictional boundaries (Fremier et al. 2015). This can be accomplished by building capacity to dynamically coordinate planning, funding, and actions among formal (counties, districts, boroughs, communities) and informal (social networks, nongovernmental organizations) governance bodies (Brondizio et al. 2009).

We use the term social connectivity (sensu Kondolf and Pinto 2017) to refer to the flow of information among institutions and knowledge across sectors (policy, research, practice) to improve understanding and governance of cross-boundary processes.

Estuaries are good examples of cross-scale dependent systems. They occur at the interface of the freshwater, marine, and terrestrial realms. This setting creates complex, dynamic, productive, and biodiverse systems affected by many interconnected forms of connectivity that vary in spatial and temporal scale. Estuaries have sustained human life since the earliest times and often are a tremendous economic resource (Thrush et al. 2013b, Fang et al. 2018). Consequently, many estuaries are altered and managed by humans (e.g., Matella and Merenlender 2015, Dettinger et al. 2016). Ecoscape connectivity of estuaries can be divided into five components: longitudinal connectivity (e.g., upstream–downstream), lateral connectivity (e.g., land–water), vertical connectivity (e.g., atmosphere–surface–groundwater), lattice connectivity (omnidirectional movements), and teleconnectivity (long-distance movements between stepping stones; figure 1, table 1). Hydrological connectivity has a major influence on the first three components but not necessarily on the latter two. Although we are focusing on the spatial aspects of connectivity, we emphasize that landscapes—and estuaries in particular—are dynamic and connectivity can change hourly, seasonally, annually, and over decades (Zeller et al. 2020).

**Table 1.** Connectivity components of an estuarine ecoscape.

Connectivity component	Definition	Connected realm	Examples of ecological connectivity	Examples of overconnectivity	Examples of barriers
Longitudinal connectivity	Directional linear movement up or downriver	Freshwater–freshwater, freshwater–marine	Salmon migration from uplands to ocean, movement of nutrients and sediment	Channelization increases water velocity	Inadequate flow, dams, diversions; elevated predation by nonnative species in migration corridor bottlenecks
Lateral connectivity	Connectivity at the land–water interface of shallow, structurally complex habitats	Freshwater–terrestrial, marine–terrestrial	Tidal marshes, riverine floodplains	Transfer of contaminants (e.g., pesticides, roadway runoff) from land to water	Levees, dikes, ditches, canals, modifications of shorelines and riverbanks
Vertical connectivity	Exchange of surface water to groundwater or marsh porewater	Freshwater–freshwater, freshwater–brackish	Long inundation periods, hyporheos	Transfer of contaminants (e.g., fertilizers, pesticides) between surface and groundwater	Inadequate flow/inundation, groundwater pumping
Lattice connectivity	Movement can be omnidirectional, but an organism must move through each adjacent lattice element to move from one location to the next	Terrestrial–terrestrial, freshwater–marine, marine–marine	Migrating and dispersing terrestrial species (except flying species), omnidirectional larval dispersal (not constrained by riverbank geometry)	Land use change of natural barriers	Linear infrastructure (e.g., roads, railways, aqueducts), development, natural barriers, habitat loss
Teleconnectivity	Organisms moving long distances without the need to move through adjacent lattice elements	All	Migrating birds, bats, and insects, plant propagule dispersal by air	Invasive species moved by human transportation, pollution	Habitat loss

In the present article, we describe ecoscape connectivity of estuaries using the example of the San Francisco Estuary, in California, in the United States. This estuary forms where the Sacramento and San Joaquin Rivers join in a freshwater inland delta and exit toward the ocean through a series of large bays with successively higher salinity. We describe the natural and altered states of this estuary and demonstrate the human dimensions of governing this complex system. We explore hydrological, ecological, and policy challenges to balance ecoscape connectivity in estuaries and provide recommendations for policy, practice, and research on how to best restore appropriate connectivity to cross-boundary systems.

## Ecological aspects of ecoscape connectivity

We recognize that the five aspects of ecoscape connectivity are not always clearly distinct from each other. However, by laying out the natural and altered states of the five spatial aspects of ecoscape connectivity in estuaries, indicating restoration options, and considering related human dimensions (table 2), we illustrate the complexity of ecoscape connectivity in cross-boundary systems.

**Table 2.** The natural and altered states of different aspects of ecoscape connectivity in estuaries, restoration options, and human dimensions.

	Natural state	Altered state	Restoring connectivity	Human dimensions	References
Longitudinal connectivity	Connected dendritic river system allows movement of aquatic species; sediment moves from the uplands and is deposited in the estuary; coherent river networks allow source water to increase in concentration for salmon homing toward spawning river; fish bring nutrients upstream	Disconnected at watershed scale: dams pose barriers to fish movement that limits available habitat for migrating fish; altered migration timing due to altered flow regime; overconnectedness in flat, estuarine areas due to channel cuts and straightening for navigation leading to homogenization of aquatic habitat; pervasive predation by invasive fish species disrupts connectivity	Fam removal; fish ladders; trucking fish; sediment bypasses; living shorelines; restoring dynamic flow regimes; blocking channels to reestablish complex channel networks; for native fish navigation, creating coherent aquatic networks offering chemical cues for migration, water velocities for energy conservation, and structural complexity for cover from predators; restoring large wetlands that can naturally develop and maintain dendritic channel networks at intervals for migrating fish to have holding and rearing habitat patches a day's travel distance apart	Importance of flood control and water supply for human communities and agriculture; tolerance for endangered species reintroductions; large-scale restoration may require a legal framework; consider existing laws and policies and legal authority to act when prioritizing conservation efforts	Lusardi and Moyle 2017, Yarnell et al. 2020, Milner et al. 2019, Vannote et al. 1980, Michel et al. 2020, Moore 2015, Schindler et al. 2005, Grill et al. 2015, Kondolf et al. 2014,
Lateral connectivity	The land–water interface is extensive in the form of tidal wetlands and floodplains; high primary production and invertebrate production on intermittent off-channel habitat and floodplains is distributed into rivers and sloughs	A loss of tidal wetlands, riparian ecosystems, and floodplains; loss of aquatic–terrestrial connectivity due to levees constructed for flood protection and reclamation; reduced primary productivity input into rivers and sloughs; influx of contaminants from agriculture and developed areas, toxic runoff from roads	Levee setbacks, breaches, or removal; tidal wetland restoration; flow alteration to restore lateral connectivity; flow management to inundate floodplains, coupled with flushing events to export food downstream; reconnecting tributaries to estuaries through wetlands; increasing fish access to floodplains; contaminant regulations	Multiple benefits of floodplains; benefits to fisheries; economic disparity	Walton et al. 2020, Moyle et al. 2004, Enright et al. 2013, Colombano et al. 2020b, 2020a, 2021, Tian et al., 2020, 2021, Herbold et al. 2014, Wang et al. 2019, Yarnell et al. 2015,
Vertical connectivity	Stable groundwater level and hyporheic exchange; upwelling	Reduced surface area for hyporheic exchange; loss of upwelling sites due to straightening of channels; drying out of floodplains due to groundwater overdraft; loss of riparian forests	Removal or set back of levees; increasing structure in channels; slowing flow to allow for more surface water–groundwater connection; stopping groundwater overdraft; increasing flooding depth by increasing flow volumes; marsh “farming” for peat accretion, and paludiculture	Trade-off between agricultural and urban areas and restored areas; trade-off between human and ecosystem use of water	Stanford and Ward 1993, Yarnell et al. 2015, Boulton et al. 2010, Vervier et al. 1992, Mount and Twiss 2005, Robinson et al. 2014,
Lattice connectivity	Terrestrial ecosystems of estuaries e.g., riparian areas, are intact, abundant, and connected to each other and to the estuary's surrounding	Terrestrial ecosystems are small and fragmented by habitat loss, levees, and roads	Strategic habitat restoration to increase connectivity; restoration of corridors (e.g., hedgerows, riparian vegetation); wildlife crossings over roads and other barriers	Community stewardship; benefits for recreation; benefits to agriculture from increase pollinator abundance; trade-off between agricultural and restored areas; costs of restoration and construction	Townsend and Masters 2015, Valerio et al. 2021, Hamilton et al. 2010, Sybertz et al. 2017,
Teleconnectivity	Great abundance of migratory species especially birds and fishes, in estuaries; complete food webs composed of only native species, including reliable abundance for migratory species	Diminished functions of estuaries due to loss of habitat due to sea level rise, invasive species, etc., reduces value as stepping stones, overwintering, or summer ranges for migratory species; estuaries can provide new habitat for species shifting their ranges poleward with climate change; nonnative species introductions	Strategic habitat restoration; flow management for ecological function; ballast water regulations and treatment; nonnative species surveillance; broadscale conservation and management networks	International jurisdictions; multiple benefits of restoration; a lack of funding for large-scale restoration; competition for fresh water; shipping regulations; economic disparity	Viana et al. 2016, Lett et al. 2020, Sommer et al. 2007, Sayles and Baggio 2017, Yarnell et al. 2020, Robalo et al. 2020,

### Longitudinal connectivity

**Figure 2.**
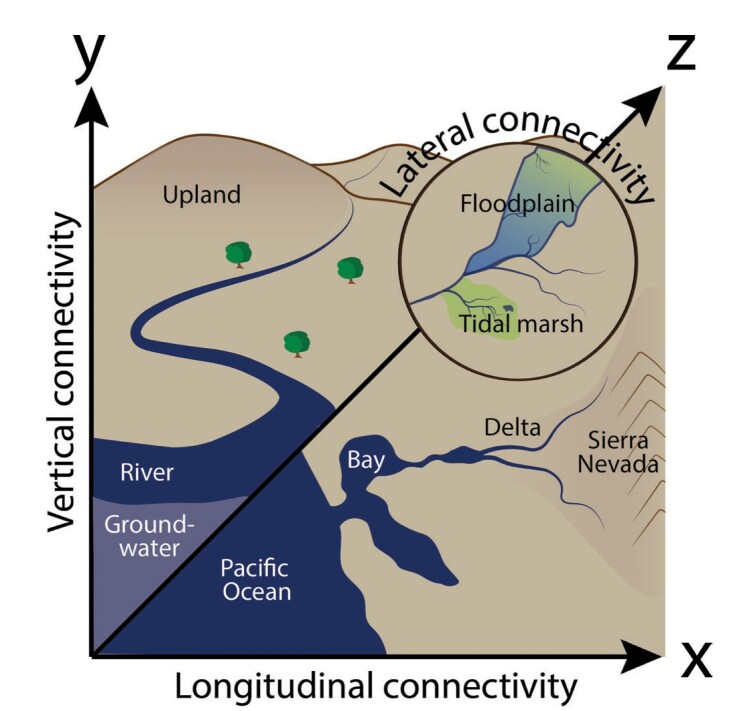
The three components of ecoscape connectivity in the San Francisco Estuary that are dominated by hydrological connectivity.

The flow of water from high elevation streams to the ocean dominates aquatic longitudinal connectivity, transporting organisms, materials, and energy and shaping biological communities (figures 1 and 2; Vannote et al. 1980). A key process supported by longitudinal connectivity is the transport of sediments, organisms, and nutrients from the uplands to the ocean. Sediment deposition creates complex, dendritic river deltas and productive, biodiverse wetlands that support an array of species and perform key ecosystem services such as flood control. Uninterrupted connectivity across the salinity gradient also enables the bidirectional movements of organisms foraging or rearing in the productive boundary zones and allows diadromous species to access both ocean and freshwater habitats (Moore 2015). Salmon spawning migrations transport marine-derived nutrients vast distances upstream, creating important connections between oceanic, terrestrial, and riverine food webs (Schindler et al. 2005). In an intact estuary, the dendritic channel system creates the habitat complexity necessary to support the diversity of behaviors and life history patterns exhibited by resident and transient species, as well as increased opportunities for exchange of materials between marine and freshwater realms.

However, longitudinal connectivity is increasingly disrupted by human activities. About half of the world's rivers are now dammed or diverted, reducing flow, altering the timing of seasonal cues, and creating physical, ecological, and hydrological barriers between the estuary and upper watershed (Grill et al. 2015, Sturrock et al. 2020). Sediment is trapped behind dams, causing deficiencies downstream and resulting in increased coastal erosion rates, habitat loss, and flood risk (Kondolf et al. 2014). Dams and other barriers (e.g., weirs, road crossings) also obstruct fish passage, eliminating swaths of ancestral habitat and contributing to drastic declines observed among many freshwater and diadromous species (Grooten and Almond 2018). Physical barriers within estuaries (e.g., tidal barriers) and loss of critical habitats (e.g., through land development and sea level rise) can impair longitudinal connectivity between marine, estuarine, and upstream areas.

At the same time, actions aimed at improving the efficiency of water conveyance and navigation in estuaries (e.g., channel cuts, straightened river channels, levee construction) can greatly increase hydrological connectivity. This can homogenize physiochemical conditions, reduce primary productivity, and eliminate low-velocity refugia needed by aquatic organisms to forage and escape predators (Safran et al. 2016). Another example of augmented connectivity in the San Francisco Estuary involves the trucking of millions of salmon smolts from upstream production hatcheries directly to downstream “bays” from which they can more easily reach the ocean (Sturrock et al. 2019). The loss of olfactory waypoints results in excessive straying rates among the returning adults, and consequently, system-wide genetic and demographic homogenization (Dedrick and Baskett 2018).

To restore longitudinal connectivity, barrier removal can be highly effective (Bednarek 2001), but is rarely implemented given the high costs, lost revenue, and potential impacts on flood control and water supply (Grantham et al. 2014). Sediment replenishment and habitat restoration can restore some aspects of natural connectivity below dams but are most effective when implemented in parallel with strategic flow management (Stähly et al. 2019, Munsch et al. 2020). Dams can be operated to mimic aspects of the natural flow regime to maintain essential biophysical processes (Yarnell et al. 2015), but this relies on sufficient water storage and allocation, increasingly an issue in regions subject to severe multiyear droughts (such as California). If the topography allows it, fish passage can also be enabled by physical structures built into or near barriers (e.g., fish ladders, culverts); otherwise, although not without disadvantages, fish can be collected and transported past physical or ecological barriers (Lusardi and Moyle 2017). Restoration of longitudinal connectivity—for example, by targeted restoration of habitats along migratory corridors—should explicitly consider scale, process and species-specific needs to optimize distance between habitat patches in a rapidly changing climate (Robinson et al. 2016).

### Lateral connectivity

The position of estuaries at the intersection of land and water creates complex, dynamic, and biologically rich transition zones in the lateral dimension (figures 1 and 2). Hydrologically connected habitats such as tidal marshes, riverine floodplains, and riparian areas facilitate the exchange of materials, energy, and organisms. However, these connections vary in frequency, amplitude, and duration as a function of the longitudinal gradient and habitat type. For example, although they are shallow, vegetated tidal marshes along shorelines are inundated daily by fresh, brackish, or saline waters, floodplains are inundated by seasonal freshwater flow pulses from adjacent rivers. Collectively, the interconnected habitat mosaics spanning the estuary support diverse physicochemical conditions, aquatic–terrestrial food web links, and interstitial spaces used by a wide variety of fish and wildlife for reproduction, foraging, and refuge from predators (e.g., Strum et al. 2017, Colombano et al. 2021).

Numerous species with complex life cycles are adapted to seasonal shifts in resource availability in these productive peripheral habitats. Sacramento splittail (Pogonichthys macrolepidotus), an endemic minnow in the San Francisco Estuary, spawns on riverine floodplains that are inundated during the winter months of wet years. In spring, juveniles move from riverine floodplains to downstream brackish tidal wetlands (Moyle et al. 2004). Nursery habitats are concentrated in tidal marshes where dendritic shallow channel networks, dense stands of emergent vegetation, and soft-bottom sediments enhance juvenile survival and recruitment (Colombano et al., 2020, 2021). Together, riverine floodplains and tidal marshes are critical habitats that support splittail populations at different life stages. These shallow wetland habitats, connected by riverine corridors, also provide important foraging (Goertler et al. 2018) and growth opportunities (Sommer et al. 2001) to outmigrating juvenile Chinook salmon (Oncorhynchus tshawytscha), particularly for the numerous small fish that leave their natal stream soon after emergence (Phillis et al. 2018).

The prevalence of human modifications of vegetated shorelines and riverbanks (e.g., via levees, dikes, ditches, canals, and other hardened surfaces) has disrupted lateral connectivity across the estuary. As a result, negative ecological consequences to fishes, particularly in the juvenile life stage, are widespread. In tidal marshes, natural variability in flooding of the marsh plain controls temperature, velocity, physical access to food and cover by fishes, and terrestrial subsidies to aquatic food webs (Enright et al. 2013, Cloern et al. 2021). In contrast, modified tidal channels adjacent to agriculture feature less variable hydrodynamics. Contaminant loading is a negative effect of lateral connectivity between human-affected areas and estuarine waters. It has the potential to harm organisms inhabiting aquatic habitats connected to sources of pollution such as agricultural runoff (e.g., fertilizers and pesticides) and surface runoff from roadways and creeks (Wang et al. 2019, Tian et al., 2020, 2021). Collectively, wetland degradation and fragmentation may scale up to population responses by Sacramento splittail and Chinook salmon because of reduced availability of spawning, foraging, and rearing habitats.

When restoring connectivity in the lateral dimension an emphasis on process is key. Tidal marsh and floodplain restoration can be achieved by breaching or removing levees while considering timing, magnitude, and duration of flow patterns to optimize desired outcomes for target species. With climate change and sea level rise, restoring tidal marshes is recognized as a critical strategy to help protect both the natural and built communities at the estuary's shore from erosion and inundation from waves, storms, and tides (Spalding et al. 2014). In the upper San Francisco Estuary, actions are also being taken to temporarily connect agricultural flood control bypasses, thereby increasing the access and capacity of remnant seasonal floodplains in supporting juvenile fishes and overwintering birds (National Marine Fisheries Service 2019, Bird et al. 2001).

### Vertical connectivity

Prior to significant modification by European settlers, the San Francisco Estuary supported low-elevation islands and a variety of wetlands around the land–water interface, including riverine floodplains and tidal marshes, all with high water tables and strong interactions between surface and shallow groundwater (figure 2; Goals Project 1999, Whipple et al. 2012). The delta was the unimpeded convergence of the lower reaches of several major rivers, with wide floodplains and shallow alluvial aquifers. During high river flows, these areas experienced deep inundation over periods of weeks or months. The lower reaches of rivers flowing into the estuary were characterized by strong vertical exchanges between surface waters and the shallow groundwater, known as the hyporheic zone. Undulations in the channel bed drive hyporheic exchange, important for nutrient exchange and creating thermal refugia for fish in sites of groundwater upwelling (Boulton et al. 2010). The hyporheic zone is recognized as an especially biodiverse and productive ecotone, which acts as a filter between groundwater and surface water (Vervier et al. 1992). Moving downstream in the estuary, mixing of brackish and saline water and tidal forcing add increasing spatial and temporal complexity to these interactions. Vertical connectivity is closely related to lateral connectivity along river channels generally, and this is especially true in estuaries, with extensive flat, low-lying lands adjacent to channels.

The vertical connectivity that characterized the San Francisco Estuary was historically disrupted in conjunction with lateral connectivity, as a result of diking, draining, and filling of wetlands, which ultimately reduced the area over which floodwaters could spread. As lower reaches of rivers were straightened and dredged, loss of bed undulations resulted in loss of upwelling sites. Facilitated by agricultural practices, the organic-rich sediments that composed the large intertidal “islands” (diked freshwater tidal marshes) dried out, oxidized, compacted, or blew away as dust, resulting in subsidence of up to 7 meters over much of the delta (Mount and Twiss 2005). Therefore, these delta “islands” are now below sea level, and pumps are required to prevent the water table from rising and waterlogging crops, keeping the water table at unnaturally deep levels and eliminating vertical connectivity between surface and shallow groundwater existing in the natural state. Formerly extensive riparian forests and tidal wetlands that had been supported by this shallow groundwater have been reduced to mostly small, isolated patches (Robinson et al. 2014).

Vertical connectivity also includes ground and surface links with the atmosphere. In the delta, the main factor of delta subsidence is the loss of peat soil by oxidation after the wetlands were drained for agriculture (Weir 1950). Oxidation processes transfer greenhouse gases (carbon dioxide, methane, water vapor) to the atmosphere (Deverel and Rojstaczer 1996, Hatala et al. 2012). Sinking deltas are a global phenomenon (Syvitski et al. 2009) and inland waters, more generally, contribute to greenhouse gas fluxes to the atmosphere (Raymond et al. 2013, Hotchkiss et al. 2015).

Approaches to restoring vertical connectivity include removing or setting back levees (also key to restoring lateral connectivity), restoring natural flow regimes that result in deeper, longer flooding, increasing structural complexity in channels to create opportunities for upwelling to allow for more surface water–groundwater connection, reversing subsidence, and controlling rates of groundwater pumping, especially in areas experiencing groundwater overdraft. In addition, changing water regimes in the delta can reduce the oxidation process to both reduce or reverse subsidence and gas transfer to the atmosphere (Hatala et al. 2012). And human created reservoirs can be managed to reduce atmospheric transfers (Deemer et al. 2016). Such restoration initiatives will need to recognize land-use trade-offs among agriculture, water resource management, urban areas, and areas of restored ecosystems, and also trade-offs between human and ecosystem use of water. Of particular importance in the context of climate change are groundwater-supported riparian forests (Rohde et al. 2021, Kibler et al. 2021) and thermal refugia offered by sites of upwelling.

### Lattice connectivity

Lattice connectivity describes omnidirectional movements by species and matter in and among adjacent lattice elements (represented by cells or polygons on maps; Urban and Keitt 2001). Unlike teleconnectivity (see below), lattice connectivity dictates that movements occur between adjacent elements, which can be terrestrial or aquatic. Lattice connectivity differs from longitudinal, lateral, and vertical connectivity in that movement can be omnidirectional and does not necessarily occur along certain dimensions. In the lattice connectivity model, the loss of key corridors can severely disrupt connectivity, and the removal of barriers has the potential to dramatically increase connectivity.

Tidal marshes, floodplain forests, and other natural riparian areas provide ideal movement corridors in estuaries for volant species with short dispersal or daily movement distances (some birds, insects, and bats) and for terrestrial wildlife. Riparian shrub and gallery forests provide diverse habitat because of the density and stature of vegetation. For example, the brush thickets in the northern San Joaquin Valley provide some of the last remaining habitat for the riparian brush rabbit, a federally and state listed endangered species (Hamilton et al. 2010).

Lattice connectivity also encompasses connectivity to adjacent ecoscapes. Because of habitat loss and fragmentation, populations of endemic tule elk (Cervus canadensis nannodes) were reduced to less than ten individuals by the late nineteenth century, and wildlife agencies have been working for decades to recover their numbers (Greco et al. 2009). Today, an isolated population resides in the central part of the San Francisco Estuary, whereas another inhabits the Inner Coast Range northwest of the estuary. Restoring connectivity between these populations would provide key links between otherwise isolated terrestrial habitats.

Restoring lattice connectivity of terrestrial components in estuaries and to adjacent regions requires strategic habitat restoration of tidal marshes, floodplain forests, other riparian areas, and upland areas, as well as providing crossing structures for wildlife over major roads and aqueducts. In addition, there is an opportunity to improve the ecological quality of linear features such as roadsides, levees, agricultural field margins, and ditches that can enhance habitat connectivity for invertebrates, birds, and other wildlife (Sybertz et al. 2017). In the Sacramento and San Joaquin Valleys, which feed into the San Francisco Estuary, farmers are supported in creating pollinator-friendly plantings along roadsides and fields margins, which has the double benefit of providing floral resources for native pollinators and enhancing pollinator services to farmers (Morandin et al., 2014, 2016). Although this approach to restoring connectivity does not require large tracts of land, the areas may require protection from pesticides. Facilitating the recovery of migratory species with regional distributions, such as tule elk, requires complex collaboration and coordination across multilevel governance structures (Sayles and Baggio 2017).

### Teleconnectivity

Estuaries function as ecological crossroads with multifaceted links to distant ecoscapes (Amezaga et al. 2002). In their natural state, estuaries support a high diversity of migratory species, many in high abundance, for critical portions of their annual cycle. However, the reduced ecological function of an estuary altered by humans can have broad impacts to both flora and fauna that spend a portion of their life cycle or annual cycle within an estuary.

The annual migrations of butterflies (Brindza et al. 2008), bats (Blakey et al. 2018), and birds (Greenberg et al. 2014), depend on reliably timed and abundant resources within both aquatic and terrestrial estuarine habitats. Animal movements also facilitate the long-distance dispersal of plant propagules and pathogens (Viana et al. 2016), in addition to transport by wind and ocean currents (Lett et al. 2020). Some species, such as western sandpipers (Calidris mauri), rely on a vast network of estuarine habitats throughout their annual cycle, including the San Francisco Estuary (Warnock and Bishop 1998), and they are particularly vulnerable to altered resources at critical stopover “refueling” sites during migration. The alteration of critical estuarine habitats in the Yellow Sea, which serve as an important staging and stopover sites for migratory shorebirds in the East Asian–Australasian Flyway, has coincided with steep declines in numerous species (Studds et al. 2017).

Human alteration of estuaries supports increasing human populations by facilitating marine transit and conveyance of freshwater to distant regions (Kennish 2002). The increased connectivity for humans to distant ecoscapes has led to the accidental or intentional introduction of nonnative species, which can have long-term and potentially irreversible impacts on estuarine ecosystems. For example, food webs in the San Francisco estuary have been significantly altered by high densities of introduced clams and piscivorous predators, reducing the success of native fish species both via bottom-up (Sommer et al. 2007) and top-down (Michel et al. 2020) mechanisms. The resulting trophic structure is less complex and seasonal abundance is less reliable than in the past (Bishop et al. 2017).

The restoration of natural processes within estuaries can reverse or mitigate anthropogenic impacts (Borja et al. 2010) and increase the estuaries’ role as stepping stones in long-distance migrations. To prevent and counteract the negative effects of invasive species from far-away regions, proactive, coordinated approaches, such as regulating ballast waters, aquaculture, and the aquarium trade, are critical. In addition, adequate resources and authority for nimble management to eradicate or control existing invasive species are important (Williams and Grosholz 2008). The recovery of migratory species with broadscale distributions, such as migratory shorebirds, will require complex collaboration and coordination across multilevel governance structures (Sayles and Baggio 2017) to ensure that natural processes are restored sufficiently at continental and hemispheric scales.

## Environmental governance and ecoscape connectivity

Multiple aspects of ecoscape connectivity span traditional scales of governance (Folke et al. 1998, Epstein et al. 2015). In environmental governance, scale mismatches can occur when the system of governance and the processes controlling the ecological system are not spatially (or temporally) aligned (e.g., see Sayles 2018). Scale mismatches can lead to challenges for governing bodies in recognizing and addressing the cumulative impacts of decisions or ineffectiveness in solving broader-scale problems (Folke et al. 2002, Pahl‐Wostl and Hare 2004, Dietz et al. 2003). Such challenges commonly occur where governmental policies apply only within certain boundaries on the landscape, objectives vary by governing body, and the nature of implementation varies (i.e., from direct agency management of protected areas to voluntary actions by individual landowners). Formal governments often lack the capacity to coordinate such diverse policies and actions at environmentally relevant scales, resulting in ecosystem degradation and, ultimately, the reduction of ecosystem services (Folke et al. 1998, Cumming et al. 2006). In the United States, for example, federal, state, county, and municipal governments each have assigned powers and responsibilities that relate to rivers, but the governing bodies at each level were not designed to manage riverine ecological processes that cross boundaries. Instead, the riverine landscape is governed in a piecemeal fashion. Many different policies that influence riverside land use and management originate at the federal, state, county or municipal level, depending on land ownership, primary uses, and the objectives of the governing body (e.g., see Stahl et al. 2020). The boundaries of implementation do not follow watershed boundaries. Therefore, ecosystem governance can become disconnected across boundaries in the governmental system.

An example of the mismatch between governance and physical processes critical for connectivity is the supply and transport of sediment through the rivers, delta, and lower estuary. This relates to longitudinal connectivity (sediment trapped behind dams), lateral connectivity (sediment facilitates wetland elevation gain with sea-level rise), and vertical connectivity (water depth is a strong control on sediment accretion). The success of the tremendous public investments in marsh restoration in San Francisco Bay (e.g., $500 million in property taxes to the San Francisco Bay Restoration Authority) is dependent on a sufficient sediment supply (SFEI 2021). Hydrodynamic modeling demonstrates that alterations, such as proposed large-scale engineered water conveyance or extensive levee failure due to an earthquake, would likely increase retention of sediments within the delta and result in less sediment reaching the lower estuary (Achete et al. 2017). Large-scale restoration planned for the delta may also have the same effect of reducing sediment supply to the lower estuary. Current governance does not adequately address these impacts, because decisions about water conveyance and levee maintenance generally are made at the state level within the context of water supply, and marsh restoration is not coordinated between the delta and San Francisco Bay. Therefore, the potential impacts of these decisions on marshes further seaward are often not considered by the various entities with jurisdiction over the different parts of the system, and no single governmental authority is responsible for management of the entire system.

Ecoscape connectivity is perhaps a step beyond simply aligning ecosystem and governance boundaries. The governance literature identifies working within and beyond mismatches in scale of ecosystem processes and governing institutions (Cosens et al., 2014, 2017). It is impossible to create an organizational structure and spatial boundary for every environmental problem, such as contemporary, future and unforeseen environmental problems. And we cannot change existing governance structures dramatically (e.g., the locations of county boundaries). Rather, we need to build capacity to coordinate overlapping modular approaches—involving formal government and nongovernmental institutions—to cooperatively govern ecoscape connectivity (box 1; Brondizio et al. 2016). This aspect of governance is termed network governance (Carlsson and Sandstrom 2007, Newig et al. 2010) and emphasizes the links among formal and informal elements of environmental governance to establish inclusive processes to identify goals and desired outcomes (Scarlett and McKinney 2016). Because connectivity is not confined by political boundaries, coordination across jurisdictions is vital. This coordination takes both time and money and should employ an inclusive and equitable approach that honors the needs and expertise of local communities as well as formal government. Laws mandating this coordination can bring government entities to the table for connectivity coordination, including entities such as transportation and water conveyance agencies that do not normally engage in conservation but whose actions can have large impacts on fish and wildlife.

**Box 1. The origin of the Delta Stewardship Council.**

The Sacramento–San Joaquin Delta has long been the center for conflicting human uses including water supply, fisheries, and agriculture. Due to the conflicting interests, making decisions around flood and salinity control, ensuring drinking water quality, regulating urbanization, and restoring the ecosystem to, among other objectives, prevent species extinctions had become contentious. When it became clear in the early 2000s that the situation was untenable, California Governor Arnold Schwarzenegger signed an executive order in 2006, creating the Delta Vision process to develop a strategic plan for resolving long-standing conflicts between water exports, ecosystem conservation, and in-delta land use. He charged the Delta Vision Blue Ribbon Task Force, a seven-member body of senior policy experts, with developing a plan over a 2-year period. During its work the task force found that over 200 federal, state, and local government agencies have some jurisdiction in the delta. This governance structure created a fragmentation of policies, making successful ecosystem restoration and ensuring a reliable water supply impossible to achieve. One of the task force recommendations was to establish a new governance structure with the authority, responsibility, accountability, science support, and secure funding to achieve the remaining Delta Vision Strategic Plan goals. In 2009, the Delta Reform Act was passed, creating the Delta Stewardship Council with the coequal goals of protecting the delta ecosystem and providing a more reliable water supply for California while recognizing and enhancing the unique cultural, recreational, and agricultural values of the California Delta as an evolving place. The jurisdiction of the Delta Stewardship Council consists of the six counties that make up the legal delta and Suisun Marsh.

Bridging organizations and social networks are key elements of network governance. They provide capacity to facilitate responses and proactive adaptation to environmental change across jurisdictional boundaries. Nongovernmental organizations and community-based organizations can be particularly effective at interacting with multiple levels of government to address environmental issues at local and regional scales. In addition, social connectivity is an important component of the implementation of policies and management actions that can be facilitated by identifying management actions with benefits to multiple forms of connectivity. If there is effective information sharing among groups, then learning from management experiments in one place can inform actions throughout the network (Newig et al. 2010).

## Discussion and recommendations

Ecoscape connectivity highlights the importance of acknowledging and understanding the multiple forms and scales of connectivity in ecosystems. Typically, a single form (e.g., sediment delivery, species movement) at a single scale and dimension (e.g., longitudinal, lateral with respect to rivers) are the focus of governance and management actions. Ecoscape connectivity emphasizes the potential synergies or impacts of governance actions across forms and scales of connectivity (e.g., what are the benefits or impacts to terrestrial connectivity if longitudinal connectivity is altered?). For this reason, the main challenge in the governance of ecoscape connectivity is communication across existing formal institutional boundaries (such as country, state, county boundaries) and integration of bridging organizations and other forms of informal governance, such as nongovernmental organizations, social networks, and communities.

Our view is that even if we understood all the biophysical and ecological processes in the multiscalar system, that understanding alone does not give us a way to mobilize actions to restore ecoscape connectivity. More specifically, scientific understanding of the ecoscape can inform governance for restoring appropriate connectivity to cross-boundary systems but cannot change legal systems themselves without political action. Best available science that assesses connectivity needs at multiple scales and in multiple dimensions (see table 1) and evaluates interactions among the different forms of connectivity is vital.

However, this is only an initial step. Governing multiple forms and scales of connectivity will require coordination of management actions across levels, sectors, and geographies. These agreements already exist in many cases (e.g., interagency coordination agreements), but to build capacity for connectivity governance they may need to be revisited and strengthened (Cosens et al. 2014). In some instances, new conversations may need to be initiated around particularly complex resource topics and new organizations may be required to support governance of cross-scale connectivity. For example, multiple large bridging organizations have formed to support cross-sector governing of aquatic systems, such as the Delta Stewardship Council (box 1) and the Watershed Councils in Oregon, in the United States, a nonregulatory group that works with various forms of government partners and private landowners to facilitate understanding and restoration of freshwater ecosystems.

Governing ecoscape connectivity requires financial and technical investment in network governance that acknowledges the existing complex legal landscape and reignites coordination among institutions, identifies coordinating gaps for bridging organizations, and incorporates social learning. Recently, private foundations have been supporting capacity for conservation of migratory species in developing nations (e.g., Kraeger 2010). These private investments have laid the foundation for formal governance structures and agreements to be integrated into conservation at hemispheric scales and potentially beyond.

### Governance recommendations

We recommend various steps for initiating network governance processes to better govern ecoscape connectivity. We provide examples from the Sacramento–San Joaquin Delta for context.

Promote conversations about ecoscape connectivity and governance. Scientific understanding of the multiple forms and scales of connectivity is fragmented across disciplines. Synthesis of connectivity concepts and empirical evidence across the ecological and physical sciences will improve our understanding of the causes and outcomes of connectivity disruptions. Setting science-based targets and monitoring progress with indicators toward meeting the targets is an effective way to achieving progress (Dey and Schweitzer 2014). Ecoscape connectivity targets would need to be set by cooperative partnerships and could be achieved through combined action of governmental, organizational, and private actors. For measuring progress towards ecoscape connectivity in the San Francisco Estuary, a set of indicators suitable for measuring the different aspects of hydrological, ecological, and social connectivity of cross-boundary systems would need to be identified. Environmental policies are commonly informed by the best available science from disciplinary perspectives but the design and implementation of policy itself could be informed by integrative conceptual approaches as presented in the present article. If incorporated into the best available science that informs policy, ecoscape connectivity could help bridge gaps in understanding and communication of effective cross-scale projects.

Identify and leverage existing coordination capacity. Government agencies focused on resource are likely already engaged in cross-boundary, cross-agency coordination in fulfilling their missions. Identifying existing coordination agreements and activities related to ecoscape connectivity can provide an assessment of coordination capacity and opportunities. Ecoscape connectivity can serve as a guiding concept to inform the implementation of existing legislation and funding mechanisms to meet ecoscape connectivity goals. Scientists can open conversations with policymakers or nongovernmental organizations about how legislation and funding mechanisms can better foster coordination across the ecoscape. One example of an emergent bridging organization that leveraged and improved coordination capacity is the Delta Stewardship Council (box 1). The Council provides coordination at the delta scale to manage conflicts in resource use across six counties in the delta.

Identify gaps in coordination, funding, and training. Application of an ecoscape connectivity conceptual model can help identify gaps where bridging actions are necessary, or science training is required. A second step would then be to identify legislative and funding opportunities to develop coordination capacity. One example of such a gap is the limited coordination of watershed processes such as sediment transport across the expanse of the Sacramento and San Joaquin basins. Given that the Delta Stewardship Council already coordinates delta science and governance, perhaps its scope could expand to include watershed governance.

Learn from other complex hydrological systems about success and challenges of network governance. Lessons can be learned by considering other systems as analogs in terms of governing connectivity. Intensive ecosystem management in the Everglades, for example, may have resulted in too rigid of a system, such that there was not enough experimentation to identify innovative solutions (Gunderson and Light 2006). Experimentation is foundational to social learning and environmental problem solving and would ideally be encouraged within an adaptive policy framework. A shared understanding of how to manage and restore connectivity may help with creating institutional flexibility that allows for adaptive management (Peat et al. 2017).

Cooperative efforts in the Chesapeake Bay have demonstrated some success in addressing water quality problems (Carey 2021) that are arguably comparable in scale to the connectivity governance challenges in the delta. Examining the successful and challenging elements of that governance system could inform plans to reenvision governance in the delta and elsewhere. In many cases, political and economic considerations constrain restoration to actions whose costs (economic and social) are lower, such as restoring lateral connectivity by breaching levees while not addressing alterations to the flow regime that will limit the effectiveness of the lateral reconnection (Kondolf et al. 2006). Evaluating the effectiveness of past conservation measures in the context of the ecoscape connectivity model may help to communicate how piecemeal efforts are inadequate to achieve conservation objectives.

### Stakeholder engagement

A major part of the solution to the challenge of ecoscape connectivity governance is to increase stakeholder engagement dramatically and effectively in conservation planning by utilizing information and communication technology breakthroughs. Web 3.0 technologies such as iNaturalist's artificial intelligence and its huge educational potential, and Web 2.0 staples such as online surveys, spatially explicit comments on maps, and knowledge graphs can be employed to significantly increase the number of people engaged in the process. Although in the typical stakeholder inclusion process a small set of representatives are included via meetings or workshops, to effectively address ecoscape connectivity all stakeholders (not just representatives) need to have the opportunity to participate. The engagement mechanisms can be designed for every level of Arnstein's (1969) ladder of participation, which ranges from the public right to know to the public participation in the final decision. In the San Francisco Estuary, scenario development with broad public participation has successfully been applied to a small region (California Department of Fish and Wildlife 2020); projects at an ecoscape scale can benefit from lessons learned at the local scale.

A separate but related approach is building scientific models in cross-disciplinary research projects or synthesizing existing ecoscape connectivity data and sharing this information on readily accessible platforms. It presents a powerful way to improve understanding, identify and refine conservation and management priorities, inform monitoring metrics, provide a tool for outreach, and coordinate governance of cross boundary systems (Stahl et al. 2020). In California, the Areas of Conservation Emphasis data set compiles connectivity maps and models that have been developed across the state into a single map framework (https://wildlife.ca.gov/Data/Analysis/Ace) that informs, for example, conservation funding priorities (Wildlife Conservation Board 2019). Similarly, the Wyoming Migration Initiative created an atlas and database of ungulate migration routes throughout the state (Kauffman et al. 2020) to inform connectivity conservation action.

Illustrating teleconnections on map platforms could provide data to support transboundary coordination and identify gaps in best available science. Governance for continental scale connectivity (spanning ecoscapes), would need to accommodate wildlife species with large home ranges, migratory movements (Barbaree et al. 2016), and projected range shifts due to climate change, as well as functionally threatened or endangered species sensitive to habitat loss or fragmentation. Teleconnectivity governance could be better informed with more specific research on long-distance connectivity for migratory species, which has become increasingly possible with new technologies (Kays et al. 2015), and furthermore, advances in the development of user-friendly resources to inform local communities, policymakers, and researchers alike. For example, recent advances in statistical modelling of community science data from eBird (www.eBird.org; Fink et al. 2019) have provided a framework for developing online, interpretive maps of species distributions (e.g., Shorebird Viz; https://ebird.org/science/status-and-trends/faq#references). Understanding commonalities between systems, learning from experimentation while building conservation and management networks, and recognizing the steppingstone importance of ecoscapes such as individual estuaries for nonnative and migratory species are key to successful ecoscape governance.

## Conclusions

Ecoscape connectivity within and among cross-scale dependent systems occurs in multiple forms, at different scales, and sometimes across different realms. In many systems, both hydrological and ecological connectivity concepts are involved. The cumulative effects of changes in different aspects of ecoscape connectivity in modified landscapes can have nonlinear responses to ecosystem function. Together with the many other examples given in the present article, the San Francisco Estuary exemplifies an ecoscape with declining biodiversity and increasingly impaired functions for both humans and other organisms. More actions are urgently needed to restore appropriate levels and types of connectivity to support desired functions, prepare for rising temperatures and sea levels, and retain ecosystem services.

Effective ecosystem governance of cross-scale dependent systems requires the recognition of the different aspects of ecoscape connectivity. However, restoring connectivity of cross-scale dependent systems will be difficult to balance with human needs. Although it can be challenging to restore natural hydrological and ecological processes in systems heavily used by humans, such as estuaries and rivers, restoring such processes can provide valuable ecosystem service benefits. For restoring connectivity to distant ecoscapes, collaboration between international jurisdictions, finding multiple-benefit solutions, and devising funding mechanisms for large-scale restoration are vital. Governance, both formal and informal, needs to be supported by social networks reaching beyond political boundaries. To restore ecoscape connectivity, we recommend increasing awareness of ecoscape connectivity and the challenges of governance and identifying and leveraging existing coordination capacity. Where coordination is lacking, securing funding, and building capacity can fill existing gaps. Although every system is unique, lessons learned about successes and challenges of network governance in other complex systems likely can provide valuable information. With new technologies, greatly increasing stakeholder engagement in conservation planning has become feasible and can be tapped for ecoscape connectivity governance.

## Author Biographical

The author order, except for the lead author, was determined using a random generator. Annika T. H. Keeley (annika.keeley@yahoo.com) was a senior environmental scientist at the Delta Stewardship Council in Sacramento, California, in the United States, and a member of the Connectivity Conservation Specialist Group of the International Union for the Conservation of Nature. She is now a senior conservation scientist with the Center for Large Landscape Conservation in Bozeman, Montana, in the United States. Blake A. Barbaree is a senior avian ecologist with Point Blue Conservation Science, based in Petaluma, California, in the United States. Samuel M. Bashevkin is a senior environmental scientist at the Delta Stewardship Council, in Sacramento, California, in the United States. Eva E. Bush is a senior environmental scientist at the Delta Stewardship Council Delta Science Program, in Sacramento, California, in the United States, and is president-elect of the California–Nevada chapter of the American Fisheries Society. Denise D. Colombano is a Delta Science Postdoctoral Fellow and fish ecologist at the University of California, Berkeley, in Berkeley, California, in the United States. Thomas E. Dilts is a spatial analyst and research scientist at the University of Nevada, Reno, in the United States. Alexander K. Fremier is an associate professor at Washington State University. John A. Gallo is senior scientist at Conservation Biology Institute, in Corvallis, Oregon, in the United States, and is a member of the Connectivity Conservation Specialist Group of the International Union for the Conservation of Nature, and director of the Link Mapper software project. Pascale A. L. Goertler is a senior environmental scientist and fisheries ecologist at the Delta Stewardship Council, Delta Science Program, in Sacramento, California, in the United States. Melanie Gogol-Prokurat is a senior environmental scientist and spatial ecologist at the California Department of Fish and Wildlife's Biogeographic Data Branch in Sacramento, California, in the United States. J. Letitia Grenier directs the Resilient Landscapes Program and is a senior scientist at the San Francisco Estuary Institute, in Richmond, California, in the United States. Patrick R. Huber is a project scientist in the Department of Environmental Science and Policy at the University of California, Davis, in Davis, California, in the United States, and is a scientific advisor to the Wildlife Corridor Working Group. Mathias Kondolf is a professor of environmental planning at the University of California, Berkeley, in Berkeley, California, in the United States. Angela Laws is an endangered species conservation biologist with The Xerces Society, in Portland, Oregon, in the United States. Amanda T. Stahl is a postdoctoral research associate at Washington State University, in Pullman, Washington, in the United States. Anna M. Sturrock is a lecturer at the University of Essex, in Colchester, England, in the United Kingdom, and is affiliated with the University of California Davis Center for Watershed Sciences and the steering group of EU COST Action CA19107: Unifying Approaches to Marine Connectivity for improved Resource Management for the Seas.
